# Spatial transcriptomics in breast cancer: providing insight into tumor heterogeneity and promoting individualized therapy

**DOI:** 10.3389/fimmu.2024.1499301

**Published:** 2024-12-19

**Authors:** Junsha An, Yajie Lu, Yuxi Chen, Yuling Chen, Zhaokai Zhou, Jianping Chen, Cheng Peng, Ruizhen Huang, Fu Peng

**Affiliations:** ^1^ West China School of Pharmacy, Sichuan University, Chengdu, China; ^2^ State Key Laboratory of Southwestern Chinese Medicine Resources, Chengdu University of Traditional Chinese Medicine, Chengdu, China; ^3^ Cardiovascular Department, Hospital of Chengdu University of Traditional Chinese Medicine, Chengdu, China; ^4^ Department of Clinical Medicine, Zhengzhou University, Zhengzhou, China; ^5^ School of Chinese Medicine, The University of Hong Kong, Hong Kong, Hong Kong SAR, China; ^6^ Key Laboratory of Drug-Targeting and Drug Delivery System of the Education Ministry, Sichuan Engineering Laboratory for Plant-Sourced Drug and Sichuan Research Center for Drug Precision Industrial Technology, Sichuan University, Chengdu, China

**Keywords:** spatial transcriptomics, tumor microenvironment, individualized precise treatment, breast cancer, heterogeneity

## Abstract

A comprehensive understanding of tumor heterogeneity, tumor microenvironment and the mechanisms of drug resistance is fundamental to advancing breast cancer research. While single-cell RNA sequencing has resolved the issue of “temporal dynamic expression” of genes at the single-cell level, the lack of spatial information still prevents us from gaining a comprehensive understanding of breast cancer. The introduction and application of spatial transcriptomics addresses this limitation. As the annual technical method of 2020, spatial transcriptomics preserves the spatial location of tissues and resolves RNA-seq data to help localize and differentiate the active expression of functional genes within a specific tissue region, enabling the study of spatial location attributes of gene locations and cellular tissue environments. In the context of breast cancer, spatial transcriptomics can assist in the identification of novel breast cancer subtypes and spatially discriminative features that show promise for individualized precise treatment. This article summarized the key technical approaches, recent advances in spatial transcriptomics and its applications in breast cancer, and discusses the limitations of current spatial transcriptomics methods and the prospects for future development, with a view to advancing the application of this technology in clinical practice.

## 1 Introduction

High-throughput sequencing (HTS), also known as next-generation sequencing (NGS), is a class of technologies that can sequence a large number of DNA or RNA samples simultaneously. Since its emergence, it has gradually become the most important tool for human genetic research due to its high sequencing efficiency, short time, low cost and ability to provide more accurate and richer genome sequence information (1). In the early development stage of HTS, it was mainly focused on DNA sequencing, especially in the field of genomics (2). With the continuous development of technology, RNA sequencing (RNA-seq) has gradually become a popular research direction, which promotes the research of transcriptomics and epigenetics by determining the sequence of RNA and analyzing the information of gene expression, transcript variants and variable splicing (3). With the rapid development of bioinformatics, materials science and computer science, RNA-seq technology has been applied at the single-cell level to analyze the transcriptome expression of a single cell, which is called single-cell RNA sequencing (scRNA-seq) (4). scRNA-seq technology can enable researchers to elucidate the ‘time-dynamic expression’ of genes at the single-cell level through the selection of samples collected at different time points, thus facilitating in-depth analysis of cellular heterogeneity and identification of different cell types (5, 6). However, the spatial information of the tissue samples is inevitably lost in the process. To address this issue, researchers have begun to focus on the spatial analysis of RNA-seq.

In 2016, the concept of spatial transcriptomics (ST) was first proposed (7). ST represents an emerging frontier technology that follows the development of scRNA-seq. It can preserve the spatial location of tissues and simultaneously analyze RNA-seq data of tissue sections to locate and distinguish the active expression of functional genes in specific tissue regions (8). As a current research hotspot of single-cellomics, ST has been rated as the annual technical method of 2020, allowing the location of genes to be assessed and the tissue environment and spatial properties of cells to be tracked (9). Various sequencing technologies including RNA-seq, scRNA-seq and ST are important tools in today’s biology and genomics research, bringing more choices to researchers. Each has its unique advantages and limitations for different research needs, as shown in **Table 1**.

**Table 1:** Comparison of the advantages and disadvantages of RNA-seq, scRNA-seq and ST techniques.

	RNA-seq	scRNA-seq	ST
**Detection content**	Average transcript information of cell populations.	Transcriptomic data of individual cells.	Gene expression data from different spatial locations.
**Sample requirements**	Large sample size, typically extraction of total RNA from tissue or cells.	Requires single cells, with high sample requirements.	Requires tissue sections, combined with high-resolution microscopy.
**Cell type resolution**	No resolution at the cell type level; provide an overall transcriptome profile.	Provide insights into the expression profiles of different cell types within a sample.	Correlate gene expression with specific tissue regions or cell types.
**Scope of application**	Suitable for gene expression analysis of large samples, overall genomic trends.	Suitable for studying cellular heterogeneity, cell type classification and differences between cell populations.	Suitable for studying the spatial distribution and functional differences of cells at different locations within tissues.
**Advantages**	1. High sensitivity, suitable for large scale gene expression analysis. 2. Data processing is relatively simple. 3. Can analyze multiple samples.	1. Can analyze cellular heterogeneity. 2. Can discover new cell types and subpopulations. 3. Can reveal details of gene expression within cells.	1. Can simultaneously acquire spatial information and transcriptomic data. 2. Suitable for studying spatial heterogeneity within tissues. 3. Highly accurate localization of gene expression.
**Disadvantages**	1. Cannot detect intercellular differences. 2. Cannot obtain spatial information. 3. Difficult to obtain detailed cell type information in highly heterogeneous samples.	1. Data analysis is complex and requires significant computing resources. 2. High cost. 3. Data processing is challenging.	1. High cost. 2. Spatial resolution may be limited. 3. Sample preparation is relatively complex and requires integration with spatial information for interpretation.
**Difficulty of data processing**	Medium, can be handled by standard RNA-seq data analysis tools.	High, involves complex processes such as single cell data normalization and denoising.	High, involves spatial data integration and transcriptome analysis with spatial localization.
**Technical developments**	Mature technology and wide range of applications.	Gradual maturity of technology, faster updating, high development potential.	Emerging technology, still in rapid development, part of the technology is still in the optimization stage.
**Suitable fields of study**	Regulation of gene expression, disease-related genes, genome-wide association analysis, *etc.*	Single cell heterogeneity research, tumor microenvironment, immune cell research, *etc.*	Tissue-specific gene expression studies, developmental biology, tumor microenvironment studies, *etc.*

Detection content: various types of gene expression data that can be detected or collected during transcriptomic studies. Sample requirements: the specific conditions and quantities of biological material needed to conduct a particular type of analysis or experiment. Cell type resolution: the level of detail at which gene expression data can be analyzed and interpreted with respect to different cell types in a biological sample, determining how precisely the data can identify and differentiate gene expression patterns specific to distinct types of cells. Scope of application: the applicability of different technical methods in genomic research helps researchers select the most suitable technology to address specific research questions. Advantages: the superiority or strengths of a particular technology or method in its application, meaning the positive benefits or characteristics it can bring. Disadvantages: the limitations or shortcomings of a particular technology or method in its application, meaning the potential negative impacts or constraints it may bring. Difficulty of data processing: the complexity of data processing, analysis and interpretation when using a particular technology or method. Technical developments: the stages a technology goes through in its research, application and development process, including its maturity, rate of change and potential for future development. Suitable fields of study: the academic fields or directions of research in which a particular technology or method is best suited for application, usually closely related to the strengths and characteristics of the technology so that its full potential can be realized.

Breast cancer is one of the most common cancers in the world, and its tumor heterogeneity and biological function require further analysis. The spatial location of cells in tissues is closely related to cell function. The tumor microenvironment (TME) of breast cancer is a highly structured ecosystem, including a variety of immune cells, cancer-associated fibroblasts (CAFs), endothelial cells and extracellular matrix (ECM) and other components (10). In the occurrence and development of breast cancer, in addition to changes in cell gene expression and function, it is often accompanied by disturbances in tissue structure and spatial location of cells (11). ST combines complex phenotypes and spatial information to quantify tumor spatial heterogeneity at the single-cell level, thus achieving new breast cancer subtype identification and spatially resolved feature analysis, which is of great significance for understanding the occurrence and development of breast cancer and providing new strategies for the treatment of breast cancer (12).

In this article, we reviewed the current methods and recent advances in ST. We also discussed the application of ST in breast cancer research, focusing on how ST can be used to study tumor heterogeneity and TME, and provide a reference for solving breast cancer drug resistance.

## 2 Technical approaches to ST

The concept of ST was originally derived fromfluorescently-labeled RNA sequences (FISH), which uses fluorescent probes in combination with targets to achieve *in-situ* detection of genes for qualitative and quantitative analysis and relative position analysis. In a broad sense, technologies that can simultaneously obtain tissue or cell transcriptomics information and spatial information are classified as ST, including the initial *in situ* imaging technology and the subsequent development of laser microdissection technology and spatial indexing technology (13, 14). After the combination of microarray technology and next-generation sequencing (NGS) technology, ST goes to the detection of a large number of spatial spot information, which greatly improves the detection efficiency. The current ST sequencing generally refers to the high-throughput ST technology based on NGS technology and microarray technology.

### 2.1 Laser capture microdissection-based approaches

To obtain spatial gene expression information, the target region can first be isolated from the sample, and then the dissected single region is placed separately in a test tube for RNA extraction and sequencing. The separation of target regions can be achieved by frozen section, RNA tomography (tomo-seq), laser capture microdissection (LCM) and other methods (15–17). Among them, LCM is more favored by researchers due to its advantages of high speed, high precision and versatility.

LCM is a technique for cutting, separating and collecting selected samples under a microscope using a micromanipulation system. It works by combining a laser with an inverted microscope and connecting it to a computer for direct visual analysis (18, 19). The combination of LCM and scRNA-seq allows RNA sequencing of individual cells or tissue regions (20). On this basis, Chen et al. constructed a geographical position sequencing (GEO-seq) method by integrating and optimizing LCM and scRNA-seq, which can obtain a small amount of cell transcriptomics information while retaining the original location information of cells, so as to achieve high-throughput gene expression (21). Geo-seq enables high-resolution ST by using a technique that tags mRNA *in situ* with spatially barcoded probes to map gene expression across tissue sections.

Additionally, transcriptomics *in vivo* analysis (TIVA) enables the examination of ST profiles in living cells. However, its analytical throughput is limited, with the capacity to analyze only a few single cells at a time (22). NICHE-seq employs photoactivation to conduct transcriptomics analysis on individual cells within a specific niche, but its applicability is limited to model organisms due to the necessity of genetic engineering (23). ProximID is another approach to examine the niche within the cellular microenvironment. It innovatively identifies protein-protein interactions within cells by using proximity labeling and high-throughput sequencing, allowing for spatially resolved mapping of protein interactions. Nevertheless, it remains constrained by the limitation of low flux (24).

**Figure 1:**
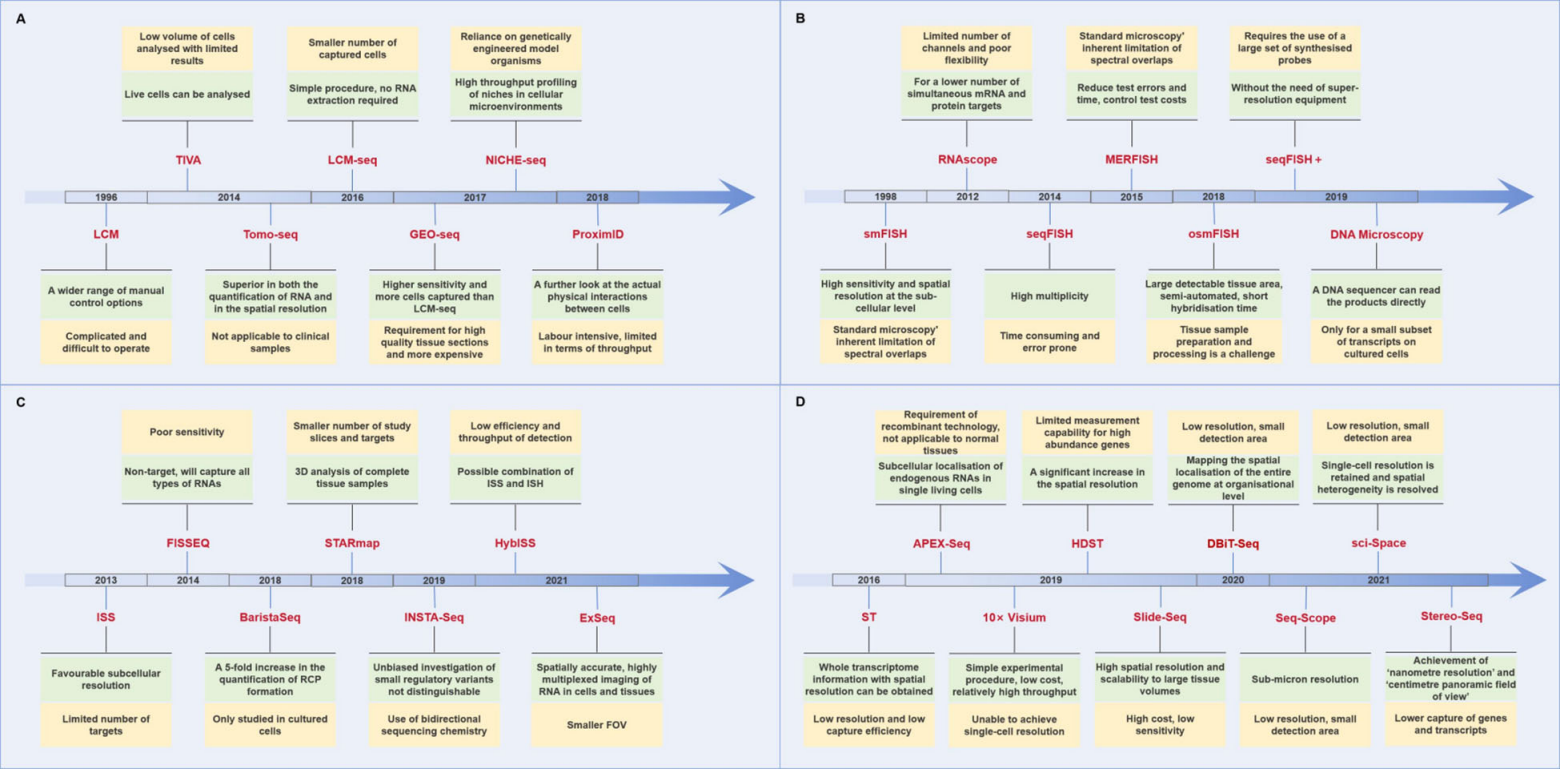
Advantages and disadvantages of different spatial transcriptomics technologies. (The green color shows the advantages of the technology, while the yellow color shows the disadvantages.) **(A)** Laser capture microdissection-based approaches. **(B)** *In situ* hybridization-based approaches. **(C)** *In situ* sequencing-based approaches. **(D)** Next-generation sequencing-based approaches.

In conclusion, as shown in **Figure 1A**, this technology can be used to obtain ST information at the cellular level; however, its analytical throughput is typically low, and the operation is complex and time-consuming.

### 2.2 *In situ* imaging-based approaches

Imaging-based ST technology employs microscopy to observe mRNA following the combination of a chromogenic group with a probe for *in situ* imaging. *In situ* hybridization (ISH) and *in situ* sequencing (ISS) are the two most commonly utilized methods.

ISH is continuously hybridized in the tissue by a marker probe that is complementary to the target sequence, and then imaged and quantitatively positioned under a microscope (25). Single-molecule RNA fluorescence *in situ* hybridization (smFISH) employs multiple shorter probes to target discrete regions of the transcriptomics, thereby enabling quantitative measurement of the transcriptomics (26). Subsequently, the advent of RNAscope technology enabled the simultaneous detection of up to 12 RNA targets, facilitating the identification of a smaller number of mRNA and protein targets (27). Continuous fluorescence *in situ* hybridization increases the signal intensity of FISH by performing multiple rounds of hybridization of a series of probes with transcripts to achieve high-throughput analysis (28–30). Furthermore, a novel technology designated as DNA Microscopy was put forth in 2019 (31). This method is based on thermodynamic entropy and does not necessitate the use of sophisticated, costly optical instrumentation. The sample itself, in conjunction with the requisite reagents, is capable of providing the requisite spatial information as part of the chemical reaction, thus assisting in the clarification of the spatial structure information present in cells and tissues (32).

In summary, ISH offers the benefits of high resolution and extensive flux detection; however, there are still constraints, including prolonged processing times, significant errors, and elevated costs (**Figure 1B**).

In 2013, the inaugural publication of ISS technology was released (33). In complete tissue sections, mRNA is initially transcribed into cDNA, and subsequently, a single-stranded DNA molecule, referred to as a lock-probe *in situ* sequencing (Lock-Seq) method, is employed to target identified genes. In comparison to ISH, ISS exhibits superior subcellular resolution; however, the number of targets that it can examine is restricted. Subsequently, FISSEQ and ExSeq enhanced the capabilities of ISS by employing two query bases to sequence circular and RCA-amplified cDNAs, as opposed to utilizing a single query base per probe to sequence gene barcodes (34, 35). The advantage of STARmap is that a second primer is added to the site next to Lock-Seq, thereby avoiding the reverse transcription step and improving efficiency and reducing noise (36). Furthermore, the advent of technologies such as BaristaSeq, INSTA-Seq, and HybISS have revolutionized ISS by improving spatial resolution, throughput, and sensitivity. BaristaSeq enables high-throughput sequencing directly from tissue sections with enhanced precision, INSTA-Seq provides rapid, single-molecule sequencing for faster analysis, and HybISS combines hybridization and ISS for precise ST (37–39). These advancements allow for more detailed, scalable, and efficient mapping of gene expression across tissues, opening new possibilities for studying complex tissue architectures and their molecular dynamics in health and disease (**Figure 1C**).

### 2.3 NGS-based approaches

The ST techniques described above are based on the separation of known tissue regions of interest, or *in situ* visualization of RNA molecules by hybridization or sequencing, while NGS technology is based on *in situ* capture of transcripts, followed by ectopic sequencing. This method was developed from the conceptual innovation of the scRNA method.

In 2016, Ståhl PL et al. successfully obtained spatial-resolved full-transcriptomics information using ST technology. This technique captures polyadenylated RNA on the slide of the spatial barcode microarray before reverse transcription, thereby ensuring that each transcript can be mapped back to its original point through a unique positional molecular barcode (40). Subsequently, this innovative approach was acquired by 10× Genomics and commercialized as a Visium space gene expression solution (41).

In 2019, high-throughput ST sequencing emerged, with the most representative method being high definition spatial transcriptomics (HDST) (42–44). HDST involves embedding silica gel magnetic beads with a diameter of 2 μm on a slide, with a specific spatial barcode connected to the surface of the magnetic beads to capture the mRNA of the cells at the corresponding position for reverse transcription and transcriptomics sequencing.

As shown in **Figure 1D**, the limitation of ST is that the analysis location is a small area of multiple cells, which is not a genuine analysis of a single cell. Consequently, subsequent new technology has focused on maintaining the resolution of a single cell or subcellular level. Seq-Scope, based on the Illumina sequencing platform, allows for the visualization of transcriptomic heterogeneity at the cellular and subcellular levels in a range of tissues, with a sub-micron resolution (45). Sci-Space combines single-cell gene expression differences with spatial backgrounds, preserving single-cell resolution (46). Furthermore, Stereo-Seq integrates DNA nanospheres (DNB) and *in situ* RNA capture technology to facilitate high-throughput transcriptomics analysis of tissue sections at unparalleled nanoscale resolutions. The visual field can be extended to the centimeter level, exhibiting high sensitivity and uniform capture rate (47). Stereo-Seq is anticipated to become a fundamental tool for analyzing transcriptomics heterogeneity in complex tissues and organisms.

### 2.4 Computer reconstruction of spatial data

The high cost of ST precludes its extensive use in clinical applications, particularly when evaluated against the backdrop of alternative experimental methods. Currently, the advent of information technology has given rise to a novel approach for calculating spatial analysis gene expression datasets. This method employs computer algorithms to simulate the spatial morphology of reconstructed tissues based on single-cell transcriptomics data. To illustrate, Seurat, an R package that is frequently employed in single-cell data analysis (48). By integrating spatial information and gene expression data, Seurat is able to reconstruct spatial transcriptional profiles of tissues, revealing the spatial distribution and functional status of cells in tissues. It can process *in situ* sequencing data of spatially tagged genes for spatial clustering, differential expression analysis and cell type annotation. In addition, Seurat supports multimodal data integration to help researchers understand the dynamics of gene expression at the spatial level and promote in-depth studies of disease mechanisms and tissue structure (49, 50).

Furthermore, the optimization of deep learning calculation methods can be conducted repeatedly in the context of continuous modeling and verification. Additionally, the prediction of ST expression can be achieved through the utilization of existing histological images (51, 52). The toolboxes Starfysh, DeepST, MISTy, standR, and others are based on deep generative models. Such methods can not only analyze the spatial dynamic characteristics of complex tissues, but also identify spatial hubs composed of different subtypes of cells. They have become invaluable for investigating the structural and functional interactions of disparate spatial backgrounds in ST.

Starfysh is designed for processing spatial transcriptome data from tissue sections. It is able to automatically identify and classify spatial regions and reveal the spatial distribution of different cell populations in tissues by combining image data and gene expression information. Using deep neural networks, Starfysh accurately combines spatial information from tissue sections with gene expression patterns to achieve a comprehensive analysis of spatial structure and function, which is widely used to study spatial heterogeneity of tissues and dynamic changes in the microenvironment (53). DeepST aims to predict the spatial location of cells in tissue sections by learning complex patterns in spatial transcriptomic data. The tool is able to automatically learn spatial associations between cells from the data by combining gene expression data with tissue section image information and using a convolutional neural network (CNN) model for spatial reconstruction and prediction. DeepST is mainly used to study complex tissue structures such as cell type distribution, spatial dynamics features, and the TME, and helps to reveal the interactions between cell function and tissue morphology (54). MISTy accurately predicts the spatial location of cells and their transcriptional status in different spatial regions by analyzing high-resolution images of tissue sections in combination with spatial transcriptome data. It can reveal the multilevel structure and dynamic changes of spatial gene expression, and is particularly suitable for spatial gene expression analysis of the tumor immune microenvironment and complex tissues (55). StandR is mainly used to solve the problem of correlation between spatial structure and gene expression of complex tissues. It is capable of identifying cell subtypes and their distribution patterns in different spatial locations through deep learning modeling of spatial transcriptome data combined with tissue section image information. standR not only supports large-scale data analysis, but also effectively identifies spatial interactions among cells, and is widely used in the study of tissue differentiation, tumor heterogeneity and disease-related microenvironments (56).

The innovative calculation method can also address the issue of ST being challenging to differentiate between similar cell populations, thereby enhancing the efficiency of sample capture. Artificial intelligence (AI) can identify different cell types and tissue regions in HE staining images and assign cell type labels to each spatial location. Combined with spatial transcriptome data, this annotation information helps to resolve the spatial layout of different cell populations in tissues and their association with gene expression (57–59). Zubair A et al. employed cell type-specific immunofluorescence markers obtained from mouse brain tissue sections and utilized artificial intelligence to annotate the output of HE tissue images, which markedly enhanced the recognition rate of clinically pertinent immune cell infiltration in tissues and broadened the scope of ST applications (60).

In short, laser capture microdissection-based approaches and *in situ* imaging-based approaches usually require expensive equipment (*e.g.* laser microdissectors, high-resolution imaging microscopes) and are therefore more costly. Both approaches also require more time for data acquisition and are less efficient, especially for complex tissue sections. NGS-based approaches and computer reconstruction of spatial data, on the other hand, are relatively less costly as they rely on high-throughput equipment and computational power, but do not require expensive hardware support. They are more efficient when dealing with large numbers of samples and can quickly process and analyze large spatial data sets. In terms of accuracy of results, laser capture microdissection-based approaches and *in situ* imaging-based approaches provide high spatial resolution and accuracy, and are suitable for fine analysis at the cellular level. NGS-based approaches and computer reconstruction, although capable of handling large data sets, may not provide the same precise spatial localization as the former two and are therefore slightly less accurate in terms of result accuracy. **Table 2** shows a comparison of the advantages and disadvantages of the different technological tools.

**Table 2:** Comparison of the advantages and disadvantages of the ST approaches.

	Laser capture microdissection-based approaches	*In situ* imaging-based approaches	NGS-based approaches	Computer reconstruction of spatial data
Advantages	1. High accurate localization of individual cells. 2. Ability to dissect and extract specific regions from tissue sections for single-cell RNA sequencing. 3. Provides a more intuitive analysis of the spatial heterogeneity in complex tissues.	1. Spatial gene expression information can be directly obtained from tissue sections, avoiding the loss caused by cell dissection. 2. Can efficiently acquire spatial distribution images and gene expression data.	1. Efficient spatial localization and indexing of large datasets. 2. Enables spatial transcriptomics analysis across the entire genome. 3. High throughput, suitable for large sample analysis.	1. Can reconstruct high-resolution spatial gene expression maps from existing low-resolution data. 2. Can integrate multimodal data from different sources. 3. Suitable for spatial reconstruction to complement existing data.
Disadvantages	1. The operation is complex and requires advanced equipment and specialized personnel. 2. Sample loss during the dissection process is relatively high, which can affect data integrity. 3. Processing speed is relatively slow, especially for large samples.	1. Imaging equipment is expensive and requires high image resolution. 2. Data volume is large, and processing and analysis require high computational power. 3. Spatial resolution may be limited by the imaging technology.	1. Spatial resolution may be lower, especially when the indexing resolution is coarse. 2. May lack precise localization at the cellular level. 3. Integration of spatial information and gene expression requires complex algorithmic support.	1. Reconstruction results are highly influenced by the quality of the original data and the precision of the algorithm.2. Requires significant computational and storage resources.3. Accuracy is limited by the reconstruction model and data resolution.
Cost	High (It requires expensive laser microdissection equipment and subsequent scRNA-seq.)	High (It requires advanced microscopy equipment, typically with complex staining steps.)	Medium (It relies on high-throughput equipment and software, relatively low cost.)	Medium (It depends on computing resources and existing data.)
Efficiency	Low (Sample processing is complex and slow.)	Medium (Imaging is fast, but data processing is large.)	High (It can handle large samples, and data processing is relatively fast.)	High (Reconstruction speed is fast, especially if based on existing data.)
Accuracy	Relatively high (It can accurately localize cells, suitable for high-precision research.)	High (It enables gene expression analysis with high spatial resolution.)	Medium (Spatial localization accuracy is lower than laser microdissection or imaging techniques.)	Medium (Reconstruction accuracy depends on data quality and algorithm precision.)

Advantages: the main advantages or strengths of different technologies and techniques used in spatial transcriptomics and related research fields, highlighting why each technique is valuable for studying spatial gene expression, tissue architecture and cellular heterogeneity. Disadvantages: the problems or limitations that a particular technology, method or process may encounter in practice, which can often affect the effectiveness, efficiency or reliability of the results. Cost: a relatively high cost associated with the technology or method, typically due to the need for expensive equipment, consumables or subsequent steps. Efficiency: the technology or method has relatively low efficiency, typically because the sample processing is complex and slow, which results in slower overall operations and difficulty in quickly handling large samples or data. Accuracy: the technology or method is capable of performing operations or measurements with a relatively high degree of accuracy, particularly in terms of accurately localizing cells or achieving the precision required for specific research purposes.

## 3 The workflow of ST

**Figure 2:**
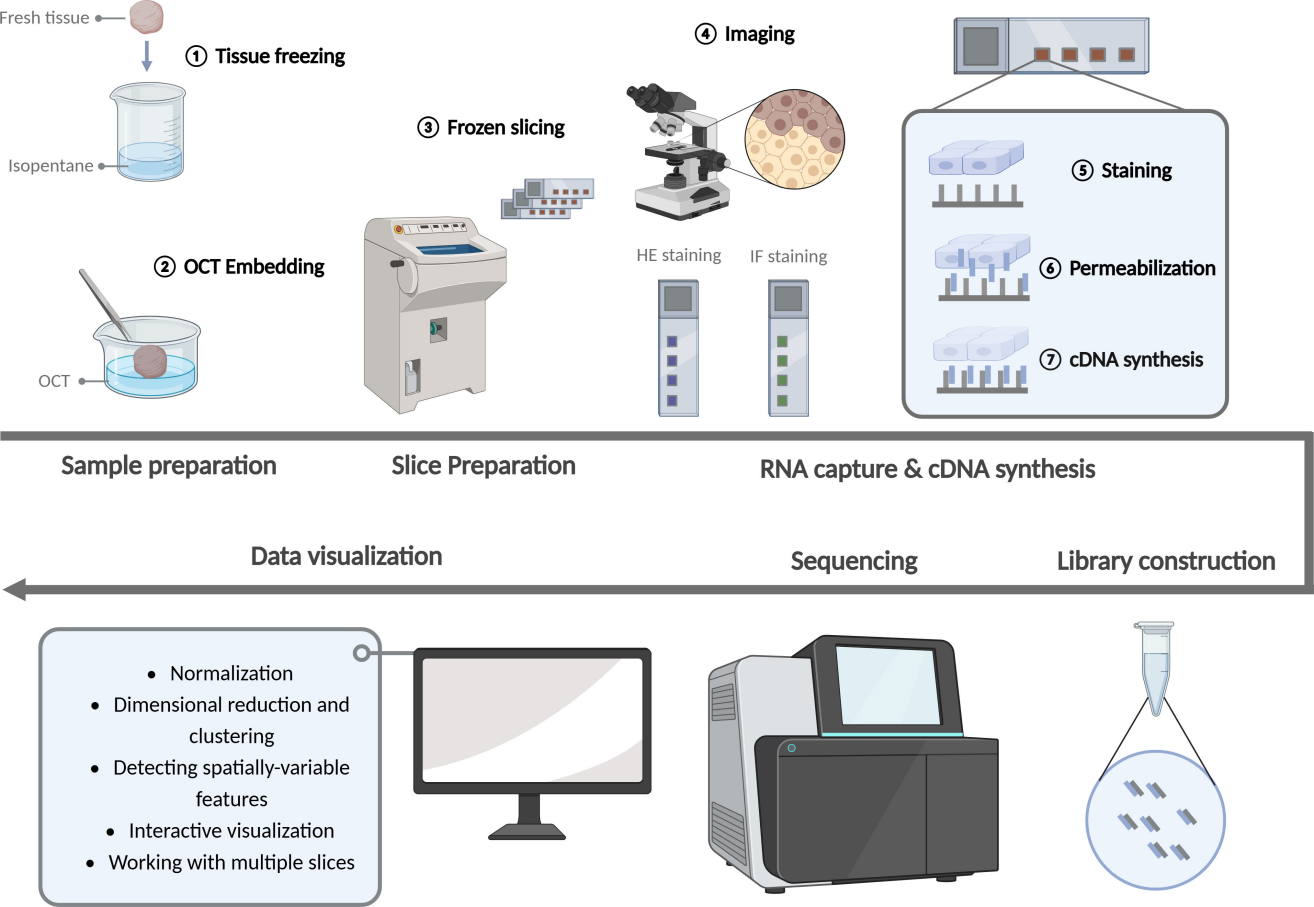
Experimentation workflow of the spatial transcriptomics. (Created with BioRender.com).

Despite the advent of numerous technical approaches to ST analysis, the majority of these methods are hindered by significant limitations, including intricate operational complexity, suboptimal detection accuracy, and constrained detection throughput. Consequently, they are not yet suitable for widespread implementation. At present, 10× Genomics Visium is the only commercially available ST sequencing scheme that offers high precision and high throughput. The technology has a straightforward workflow, a relatively short processing time, and a wide range of potential applications. The key steps in the Visium experiment are illustrated in **Figure 2**.

### 3.1 Sample and slice preparation

Initially, fresh tissue samples were fixed in isopentane and frozen in liquid nitrogen. This process was employed to safeguard the quality of RNA and the integrity of tissue morphology, thereby ensuring an accurate reflection of cellular and genetic distribution in space. Optimal cutting temperature compound (OCT) is a fixative agent. Following rapid freezing, the tissue samples must be embedded with OCT embedding agent. This has the dual benefit of reducing wrinkles and fragmentation during slicing and increasing the continuity of tissues. Prior to slicing with a freezing microtome, the tissue and 10 × slide were placed in a −20°C freezing microtome box for approximately 30 minutes. The thickness of the slice was typically 10 μm, with a maximum size limitation (61).

### 3.2 RNA capture and cDNA synthesis

After the preparation of the slices, HE staining or IF staining was performed and photographed to detect the quality of the slices and patches. Subsequently, tissue permeabilization was performed and fluorescent cDNA was synthesized. The optimal permeabilization time was then identified according to the fluorescence intensity. The slices were then processed according to the optimal permeabilization time. The addition of permeabilization reagents to the slices resulted in the mRNA of the cells being released vertically down to the slide and bound to the capture sequence of the spatial microarray. cDNA was then synthesized by reverse transcription of the captured mRNA.

### 3.3 Library construction, high-throughput sequencing and data analysis

Following the acquisition of cDNA, the library was constructed and sequenced using an illumina high-throughput sequencing instrument, thereby obtaining the requisite sequencing data. The fundamental aspect of ST data analysis is the implementation of dimensionality reduction and clustering techniques, which are based on the gene expression variations observed across different spatial units on each chip. This is then correlated with the corresponding tissue image. Concurrently, the spatial position of each gene expression within the tissue can be determined.

In general, Seurat is employed for the analysis, visualization and integration of spatial data sets. The field of spatial analysis encompasses a range of techniques, including normalization, dimensional reduction and clustering, spatially-variable features, interactive visualization, and working with multiple slices. The steps of ST data preprocessing are analogous to those of typical scRNA-seq. To address the discrepancy in sequencing depth between data points, the data must be normalized. In contrast to scRNA-seq, the molecular plot of a spatial dataset can exhibit considerable variation, particularly in instances where there are notable differences in cell density across the tissue, resulting in a substantial degree of heterogeneity. Sctransform facilitates the normalization of data, enabling the detection of high variation features and the storage of data in SCT format (62).

### 3.4 Integration of ST and scRNA-seq data

10× Genomics Visium technology is a powerful ST tool that enables efficient spatial localization of gene expression in tissue sections (63, 64). However, there are some potential limitations of this technology, especially when dealing with different types of breast cancer samples. Firstly, the limited spatial resolution of the Visium technique, typically 55 µm, means that it may not be able to capture fine-scale differences between cellular subpopulations in the TME, particularly interactions between tumor cells and immune or stromal cells. Secondly, Visium is dependent on the quality and integrity of the tissue sections, which may affect the accuracy of the data for some breast cancer samples, especially those with high tissue stiffness or heterogeneity. In addition, the technique may be affected by background noise and technical bias when processing complex tumor samples, requiring multiple optimizations and algorithmic adjustments to improve data availability and resolution. Therefore, the Visium technology still needs to be combined with other technologies (*e.g.*, laser microdissection or scRNA-seq) to obtain more accurate spatial transcriptomic data in studies of different breast cancer subtypes (65, 66).

By anchoring and integrating ST and scRNA-seq data, a three-dimensional transcriptomics map of the target tissue can be obtained. In this process, since the noise patterns of the representation space and the single-cell data set are very different, it is generally recommended to use the integral method rather than the deconvolution method (67).

The emerging ST technology, combined with microscopic imaging and scRNA-seq, can reveal the gene expression and arrangement distribution of individual cells at subcellular spatial resolution. This allows for the maximization of cellular heterogeneity, the discovery of new cell populations and cell subsets, and the clarification of the regulatory mechanisms that govern cellular status. This, in turn, provides technical support for a range of applications (68, 69).

## 4 Application of ST in the treatment of breast cancer

Conventional scRNA-seq fails to provide spatial information about cells in tumor tissue, which is particularly critical for breast cancer treatment (70). Tumor heterogeneity and microenvironment have a significant impact on treatment response, and single-cell data cannot reveal gene expression differences and cellular interactions between different regions (71). The application of spatial transcriptome sequencing in breast cancer provides an important tool to study the TME and its interaction with tumor cells. By combining gene expression and spatial information, this technology can reveal the spatial distribution and functional status of different cell populations within a tumor, helping researchers to understand tumor heterogeneity, immune escape mechanisms, and the process of tumor progression. Spatial transcriptomic data in breast cancer samples can help identify changes in immune cells, stromal cells, and blood vessels around the tumor, providing targets for precision therapy. For example, it has been found that the spatial relationship between immune cells and tumor cells may influence the tumor’s ability to escape immunity, and thus the response to treatment (72, 73). In addition, the spatial transcriptome could be used to monitor molecular signatures in different subtypes of breast cancer, which in turn could facilitate the development of personalized treatment strategies.

### 4.1 Tumor heterogeneity

Tumor heterogeneity refers to the changes in molecular biology or genes during tumor growth, resulting in differences in growth rate, invasion ability, drug sensitivity and prognosis of different tumor cells (74, 75). Despite the prevailing view that breast cancer has a monoclonal origin, the occurrence and development of this disease is the result of numerous divisions and proliferations, continuous evolution, epigenetics, genomics and microenvironment changes, which collectively give rise to heterogeneity (76). Heterogeneity represents a significant challenge in the treatment of breast cancer, manifesting as diverse histological subtypes, varying treatment sensitivities, and disparate clinical outcomes among patients. ST offers a valuable avenue for elucidating the genetic and phenotypic distinctions of tumor cells, analyzing the spatial heterogeneity of gene expression across diverse tissue regions, and providing a multi-dimensional and comprehensive perspective for investigating tumor heterogeneity (77–79).

The majority of studies have integrated ST with pathological annotation and deconvolution, utilizing diverse methodologies to accurately delineate the location, enrichment characteristics and differentially expressed genes (DEGs) of breast cancer subtypes, thereby elucidating the spatial distribution and transcriptional heterogeneity of breast cancer (80, 81). In 2013, the heterogeneity of 31 gene expression in breast cancer tissues was first observed using ISS technology. On this basis, Svedlund et al. conducted a combined molecular and morphological analysis of the expression of 91 genes in breast cancer tissues, revealing the intratumoral heterogeneity associated with breast cancer subtypes (82). Single-cell transcriptome sequencing of 26 primary breast cancers and spatial transcriptome sequencing of six breast cancers were performed to construct single-cell and spatial maps of breast cancers, revealing cellular heterogeneity in recurrent tumors and identifying novel PD-L1/PD-L2+ macrophage populations associated with clinical outcomes. Through this integrative analysis, the researchers were able to classify breast cancers into nine ‘ecotypes’ with distinct cellular composition and clinical outcomes, suggesting that the use of ST is statistically significant in classifying breast cancers and predicting response to therapy (83). A paucity of zinc finger protein 689 (ZNF689) has been demonstrated to facilitate LINE-1 reverse transcription, thereby exacerbating genomic instability and resulting in elevated intratumoral heterogeneity (ITH) (84).

The combined analysis of ST and single-cell omics allows for the consideration of both resolution and gene-level flux, which is an essential method for elucidating tumor heterogeneity (85). Studies have found that ER+ breast cancer has four heterogeneous groups, namely estrogen-responsive group, proliferative group, hypoxia-inducible group and inflammation-related group. The proliferative compartment has been identified as a key factor in the invasiveness of luminal B breast cancer (86). Liu et al. not only determined the heterogeneity of characteristics, origin and function of different cell populations in breast cancer tissues, but also employed the spatial distribution map of tumor subsets to describe the different stromal cell types in different tissue regions, providing a comprehensive method for describing the heterogeneity and structure of breast cancer (87). In luminal epithelial cells, Kohler et al. observed 11 distinct breast cancer clusters and differentiation trajectories originating from keratin 15+ (K15+) progenitor cells, indicating a robust correlation between normal K15+ primordium and basal-like breast cancer (88).

Copy number variation (CNV) can be used to reveal precancerous lesions (89). Lu et al. employed a combination of FISH and LCM with Smart-3SEQ to ascertain that HER2-amplified ductal carcinoma *in situ* (DCIS) has multiple subclones with different CNVs (90).

In addition, the spatial heterogeneity of metabolic structure is also an important part of tumor heterogeneity. ST studies have shown that glycolysis, profibrotic states, and metabolic reprogramming based on lipid metabolism play an important role in the extensive heterogeneity of breast cancer (91–93).

The high cost of ST limits its clinical application. To address this issue, some more cost-effective new methods have emerged. The deep learning algorithms ST-Net and BrST-Net can link the cell morphological characteristics and gene expression in ST, predict gene expression using HE tissue case images, and identify the heterogeneity of gene expression in tumors (94, 95). Based on the optimization and verification of a single deep convolutional neural network, the expression-morphology (EMO) analysis of the transcriptomics coverage area was established to predict gene transcription differences and proliferation markers in different regions of H&E staining images. The new method is reliable, economical and scalable (96).

**Table 3** provides an overview of studies on the use of ST to study heterogeneity in breast cancer.

**Table 3:** Summary of studies on applications of ST to breast cancer heterogeneity.

Technique	Sample	Conclusion	Reference
10× Visium, combined pathological annotations and deconvolution approaches	Fresh frozen TNBC samples with paired whole exome sequencing (WES) data	Existing bulk expression signatures of highly plastic breast cancers are relevant in mesenchymal transdifferentiated compartments, but may be confounded by abundant stromal cells in tumor samples.	(80)
10× Visium	De-identified human breast tissues and patient’s primary TNBC tumors	There is a conserved spatio-transcriptional architecture in TNBC, despite intratumoral and stromal heterogeneity within individual samples.	(81)
ISS-based OncoMaps	Fresh frozen tissue sections from breast cancer tumors	An analytical method, OncoMaps, has been developed that couples highly multiplexed gene expression profiling to the morphological features of the tumor tissue, and can deliver corresponding gene expression information on system level to clinically relevant tumor regions.	(82)
10× Visium	The multi-omics TNBC dataset from FUSCC, TNBC cases from the TCGA, and multiple mouse models of patient-derived xenograft (PDX)	In TNBC, ZNF689 has been shown to functionally modulate LINE-1 retrotransposition to reduce intratumor heterogeneity.	(84)
10× Visium and Xenium	Paraffin-embedded breast cancer tissue block	Investigation of cell neighbors and identification of rare boundary cells that are at the critical myoepithelial border confining the spread of malignant cells.	(85)
10× Visium	PDX models with distinct biological responses to oestrogen	Four active populations (i.e. oestrogen-responsive, proliferative, hypoxia-induced and inflammation-related) and a cell cluster associated with oestrogen-dependent tumor proliferation in ER breast cancer were identified, and this cell cluster is spatially and functionally distinct from cells expressing typical estrogen-responsive genes.	(86)
snRNA-seq and ST datasets	Fresh samples from primary tumors	The spatial distribution of the primary non-tumor cells was discerned in the ST dataset, indicating that neutrophils were predominantly concentrated in the luminal region, whereas B cells exhibited a propensity to infiltrate the basal region.	(87)
scRNA-seq and multicolor imaging	Normal breast tissue and breast carcinoma specimens from women undergoing mastectomy for primary breast cancer	There is a strong correlation between normal ductal progenitors and basal-like breast cancer, and K15 basal-like breast cancers originate in ductal progenitors.	(88)
Constructing a classifier and a spectral co-clustering algorithm to define biclusters	Patients undergoing mastectomy for carcinoma	Tumor initiation may not be driven by copy number aberrations and that expression data points to an altered field surrounding the tumors.	(89)
HER2-FISH and LCM Smart-3SEQ	DCIS and normal breast samples	HER2 amplification in DCIS alters the transcriptomic profiles and increases diversity of CNVs.	(90)
10× Visium	Fresh surgical specimens (primary tumors and paired metastatic lymph nodes)	A switch between glycolysis and OXPHOS in breast cancer cells is the early event in lymph node metastasis.	(93)
10× Visium	Freshly frozen IMPC samples from breast cancer patients undergoing modified radical mastectomy	IMPC has higher levels of lipid metabolism and higher levels of SREBF1 gene expression.	(91)
10× Visium and ST-Net	A new dataset consisting of 23 patients with breast cancer	ST-Net links gene expression with visual features in cell morphology, and infers the spatially resolved expression of key cancer-related genes from tissue images.	(94)
10× Visium and BrST-Net	The publicly accessible ST dataset	In comparison to ST-Net16, BrST-Net framework improves the gene expression prediction performance quite substantially.	(95)
Transcriptome-wide EMO analysis using large-scale deep learning and routine H&E WSIs	Three data sources consists of female patients with breast cancer	Deep learning-based image analysis for prediction of the tumor average expression of a substantial number of transcripts is possible and feasible.	(96)

### 4.2 Tumor microenvironment

Intratumoral heterogeneity emphasizes the difference of cells or cell subsets in TME, which can reshape the occurrence, development, invasion, metastasis and drug sensitivity of tumors (97). TME is constituted of a heterogeneous population of cells, including tumor cells and surrounding immune cells, interstitial cells, and active mediators (98). It can be divided into two distinct categories: an immune microenvironment, which is dominated by immune cells, and a non-immune microenvironment, which is dominated by fibroblasts. Currently, cancer research and treatment have changed from a cancer-centric model to a tumor microenvironment-centric model. ST technology is instrumental in elucidating the diversity and intricacies of cell populations within the breast cancer microenvironment, and has become a powerful tool for a comprehensive investigation of the occurrence, progression and escape mechanisms of breast cancer (99–101).

Through spatial analysis of samples from HER2+ breast cancer patients before and after neoadjuvant HER2-targeted therapy, it was found that treatment resulted in decreased activation of HER2-dependent signaling, increased immune infiltration, and that increased expression of CD45 at the time of treatment more highly accurately predicted the presence of a pathological complete remission (pCR) in patients, findings which reveal the role of ST in predicting treatment response and guide clinical management (102).

Tumor-infiltrating macrophages play a crucial role in tumor progression, and their accumulation in TME is associated with poor prognosis and drug resistance in a multitude of cancers. In TME, macrophages exhibit different phenotypic alterations, leading to heterogeneous immune activation (103). At present, the treatment of macrophages focuses on inhibiting their recruitment or reprogramming their phenotype from M2-like to M1-like. The development of ST has improved the understanding of the macrophage body, phenotype and functional plasticity, and provided a new way for macrophage targeted therapy (104). In order to study the determinants of functional heterogeneity of TME macrophages, researchers employed an *in vitro* organ-type TME (oTME) model, an *in vivo* mouse model, and human samples of breast cancer. The study found a subset of F4/80^high^Sca-1+ self-renewing macrophages mediated by Notch4 and maintained by type I interferon (IFN), which is closely related to the growth and metastasis of breast cancer (105). In breast cancer cell-infiltrating lymph nodes, macrophages are abundant in pathways that promote tumor growth, NF-κB and NOD-like receptor signaling pathways, and elucidate the differentiation trajectory of macrophages from active chemokine production to active lymphocyte activation (106). Tzeng et al. found that LSM1, a part of the cytoplasmic protein complex Lsm1-7-Pat1, affects the energy metabolism of TME macrophages and breast cancer metastasis, suggesting its potential as a diagnostic and prognostic marker (107).

Dysregulation of lipid metabolism is also a feature of TME, especially excessive free fatty acids (FFA). Studies have found that lipid-associated macrophages (LAMs), a unique macrophage subset, can highly express macrophage markers, lipid metabolism genes and lipid receptors, have M2 macrophage phagocytosis characteristics, and can enhance lipid accumulation (108). In addition, macrophages exhibit strong tryptophan metabolic activity in breast cancer, which can promote the polarization of M1 macrophages and predict the efficacy of immunotherapy (109).

The spatial distribution of tumor infiltrating lymphocytes (TILs) can be a predictor of the prognosis of breast cancer and the response to systemic treatment. Romanens et al. employed ST-FFE to analyze the immune cell subsets in the stromal and intraepithelial regions of TNBC. Their findings indicated that, in comparison to stromal cells, intraepithelial T cells and B cells exhibited reduced diversity and stronger clonality (110).

Cancer-associated fibroblasts (CAFs) are another diverse cell population indispensable for remodeling TME. Studies have identified four distinct functional fibroblast subsets and characterized their spatial distribution patterns. Among these, iCAFs are enriched in breast cancer and play a pivotal role in promoting an immunosuppressive microenvironment, which is altered in accordance with the status of breast cancer (111, 112). In a study by Kang et al., the impact of homologous recombination defect (HRD) on TME development at varying scales was investigated. The findings revealed that HRD alters the dominant phenotype of CAFs from mCAFs to iCAFs and is associated with immune cell function (113).

**Table 4** presents a summary of studies on the applications of ST to breast cancer microenvironment.

**Table 4:** Summary of studies on applications of ST to breast cancer tumor microenvironment.

Technique	Sample	Conclusion	Reference
Unsupervised clustering analysis	The spatially resolved transcriptomic data and images of breast cancer patients from GEO database	Compared with the number in KLF5high tumors, the number of CD4+ and CD8+ T lymphocytes was significantly increased in samples from KLF5low tumors, whereas the percentage of monocytes was markedly reduced	(101)
GeoMx DSP	Samples of patients with HER2+ breast cancer	Stromal and tumor localized immune cells in the TME are more active in primary versus metastatic disease.	(100)
10× Visium	Patient’s primary breast cancer tumors	Activated CD8+ cells shows higher tumor spatial specificity than naive CD8+ cells, consistent with their antitumor function.	(103)
10× Visium	Human breast tissue samples	The spatial localization of TME macrophages impacted not only their self-renewal capacity, but also their broader phenotype.	(105)
10× Visium	Positive lymph nodes of breast cancer patients	OGCs are scattered among metastatic breast cancer cells.	(106)
10× Visium	ST data from a previous investigation	LSM1 links to macrophage, and its alterations may drive breast cancer progression.	(107)
10× Visium	Public spatially transcriptomics data	The function and spatial distribution of TAMs present an obvious heterogeneity in the TAME of breast cancer.	(108)
ST-FFPE	FFPE and fresh-frozen samples of TNBC patients	There is a highly variable spatial distribution of immune cell subsets among tumors.	(110)
10× Visium	Publicly available ST dataset	iCAFs can promote breast cancer cell proliferation, EMT and the establishment of an immunosuppressive microenvironment.	(112)
10× Visium	Human breast tissue samples	The spatial organization of the BC TME is described, focusing on the diversity and plasticity of the FAP+ CAF and its interaction with surrounding cells.	(111)
10× Visium	Publicly available ST dataset	HRD reprograms the predominant phenotype of CAFs from myofibroblastic CAFs to inflammatory-like CAFs.	(113)

### 4.3 Drug resistance

Drug resistance is the main obstacle to the treatment of breast cancer, which is related to tumor heterogeneity. Using ST to detect drug resistance-related cell subsets and explore their unique gene expression patterns is essential for predicting and reversing drug resistance. Basal epithelial subsets are located in the matrix and have the characteristics of drug resistance, and the up-regulated ACTN1 can promote chemotherapy resistance (114). MCU, as a diagnostic biomarker for breast cancer, is associated with advanced clinical status and a low overall survival rate. Inactivation of this protein has been shown to increase sensitivity to specific small molecule drugs (115).

The objective of immunotherapy is to reinstate a normal anti-tumor immune response by reactivating and sustaining the tumor-immune cycle, thereby controlling and clarifying tumors. Immunotherapy includes immune checkpoint inhibitors, therapeutic antibodies, and small molecule inhibitors. In recent years, immunotherapy has also shown robust anti-tumor activity in breast cancer, bringing long-term clinical benefits to some patients (116). Unfortunately, due to the loss of immune response and the emergence of drug resistance, this treatment is only effective for some patients. ST can facilitate a deeper understanding of the immunobiology of TME and identify biomarkers for predicting the efficacy of immunotherapy in patients (117). Tashireva et al. observed that PD-L1-positive tumors lack direct contact between PD1 receptor-expressing cells and PD-L1 ligand-expressing cells, and the lack of this specific immune response may be the reason for the low sensitivity of TNBC patients to immune checkpoint inhibitors (ICIs) (118). Indeed, only a single subgroup of TNBC exhibits responsiveness to ICIs. Investigations have examined the correlation between the spatial organization of immune cells in TNBC and T cell evasion, identifying that deposits of collagen-10, enhanced glycolysis, and activation of TGF-β/VEGF pathways are associated with resistance to anti-PD1 (119). TNBC patients with high Treg infiltration-related scores lack the stimulation of immune activation pathways, which results in resistance to immunotherapy against PD-1 (120).

OTUD4/CD73 proteolytic axis therapy represents a promising avenue for the treatment of immunosuppressive TNBC. The ubiquitination of CD73 by OTUD4 can counteract the deubiquitination of CD73 by TRIM21, resulting in the stability of CD73 and the prevention of a tumor immune response. ST80, a novel inhibitor that destroys the proteolytic interaction between OTUD4 and CD73, has the potential to enhance the efficacy of PD-L1 treatment in TNBC (121).

IMM2902 is an anti-CD47/HER2 bispecific antibody that has been developed for the treatment of patients with trastuzumab-resistant breast cancer. Furthermore, IMM2902 can also stimulate macrophages to produce CXCL9 and CXCL10, thereby increasing the level of T cells and NK cells at the tumor site (122).

Antibody-drug conjugates (ADCs) are a type of targeted therapy for cancer that connect antibodies to drugs through precisely designed chemical ligation methods, showing excellent efficacy in the treatment of breast cancer. ADCs hinder the progression of cell cycle. Cetuximab-CDK inhibitors can use the overexpression of epidermal growth factor receptor and the disordered cell cycle in invasive and drug-resistant tumors to target and effectively deliver drugs targeting cell cycle to basal-like/TNBCs, bringing new hope for the treatment of drug-resistant breast cancer patients (123).

A summary of studies applying ST to breast cancer drug resistance is presented in **Table 5**.

**Table 5:** Summary of studies on applications of ST to drug resistance in breast cancer.

Technique	Sample	Conclusion	Reference
10× Visium	Spatial transcriptomic data previously acquired	The observed association between elevated MCU expression and poor prognosis in breast cancer suggests potential impacts on the TME and T cell infiltration.	(115)
snRNA-seq and ST	Spatial transcriptomic data previously acquired	High expression of CXCL12 was linked with a prolonged survival in breast cancer.	(117)
10× Visium	FFPE TNBC samples from patients	There are notable differences between the TME profiles of PD-L1-negative and PD-L1-positive breast cancers, and the cellular composition of the microenvironment is contingent upon the nearness to the tumor cells.	(118)
10× Visium	Node negative, primary TNBC and BC from patients; metastatic TNBC; primary BC with RNAseq and WGS data	Analysis of TNBC spatial immune cell structure to measure the prognosis of various cancers and the anti-PD1 response in patients with metastatic TNBC.	(119)
10× Visium	Spatial transcriptomic data previously acquired	TK1 positive cells mainly localize in tumor area, and Treg cell infiltration in TNBC tissues was associated with high expression of TK1.	(120)
GeoMx DSP	Tumor tissue sections from patients with TNBC	Ubl conjugation, TGF-β receptor activity, and SMAD binding pathways pertaining to CD73 PTM modulation are particularly enriched in OTUD4lo compared with OTUD4hi tumor areas.	(121)
10× Visium	CB-humanized HCC1954 mouse model	The IMM2902-treated group exhibited significantly elevated levels of CD68, CD11C, CD8, and NKG7, enhancing immune cells infiltration in tumors.	(122)
10× Visium	Spatial transcriptomic data previously acquired	The cetuximab-CDK inhibitor ADC may provide a selective and highly potent cell cycle-targeting agent for basal-like/TNBC, including chemotherapy-resistant residual disease, by taking advantage of the overexpression of epidermal growth factor receptor and cell cycle dysregulation in aggressive and refractory tumors.	(123)

## 5 New hope for individualized precise treatment

Breast cancer has a variety of subtypes, which are different in genetics, histology and clinical features, and there are potential variations between different subtypes (124, 125). The treatment of breast cancer is dependent on a number of factors, including the specific subtype of cancer, the extent to which it has spread from the breast to the lymph nodes (stage II or III) or other parts of the body (stage IV), the patient’s response to previous treatment, the molecular composition of tumor cells, and the state of the patients’ immune system (126). As research progresses, the treatment of breast cancer has completed the transformation from single therapy to comprehensive therapy, and its treatment methods are also emerging in an endless stream, including surgery, chemotherapy, targeted therapy, endocrine therapy, immunotherapy and so on (127). Nevertheless, the question of how to ascertain the most appropriate treatment for each patient, taking into account their individual circumstances, has remained a significant challenge in the research and development of anti-tumor drugs and clinical treatment (128).

The ST sequencing analysis of breast cancer patients can better determine the differences in tumor heterogeneity and TME in patients with different disease stages and molecular typing, and can enable a comprehensive investigation of the diverse cell types and their molecular mechanisms *in vivo*. On this basis, targeted markers and new therapeutic targets can be determined, thereby enabling the personalized and precise treatment of cancer patients (129). For instance, some researchers have proposed a model that can reproduce the clinical treatment response. By comparing patients with pCR and no response progression (pNR), they identified differences in transcription procedures in tumors, stroma and immune infiltration, providing new ideas for the improvement and personalization of TNBC treatment methods (130).

In addition, it is important to identify the most efficacious method of eliciting anti-tumor immune responses, thereby providing patients with a more robust and effective immunotherapy. Shiao SL et al. analyzed TNBC biopsy tissue by integrating scRNA-seq and ST, and found that patients exhibiting resistance to immunotherapy exhibited a lack of immune infiltration both before and after treatment. Conversely, patients who responded to immunotherapy demonstrated two distinct patterns: one with a pre-existing immune response and the other with an immune response only evident following αPD1 and radiotherapy (131). At the same time, research has revealed that quiescent cancer cells (QCCs) in primary TNBC establish a micro-network with damaged dendritic cells, which can resist the attack of T cells, reduce immune infiltration, and demonstrate heightened tumorigenic potential (132).

**Figure 3:**
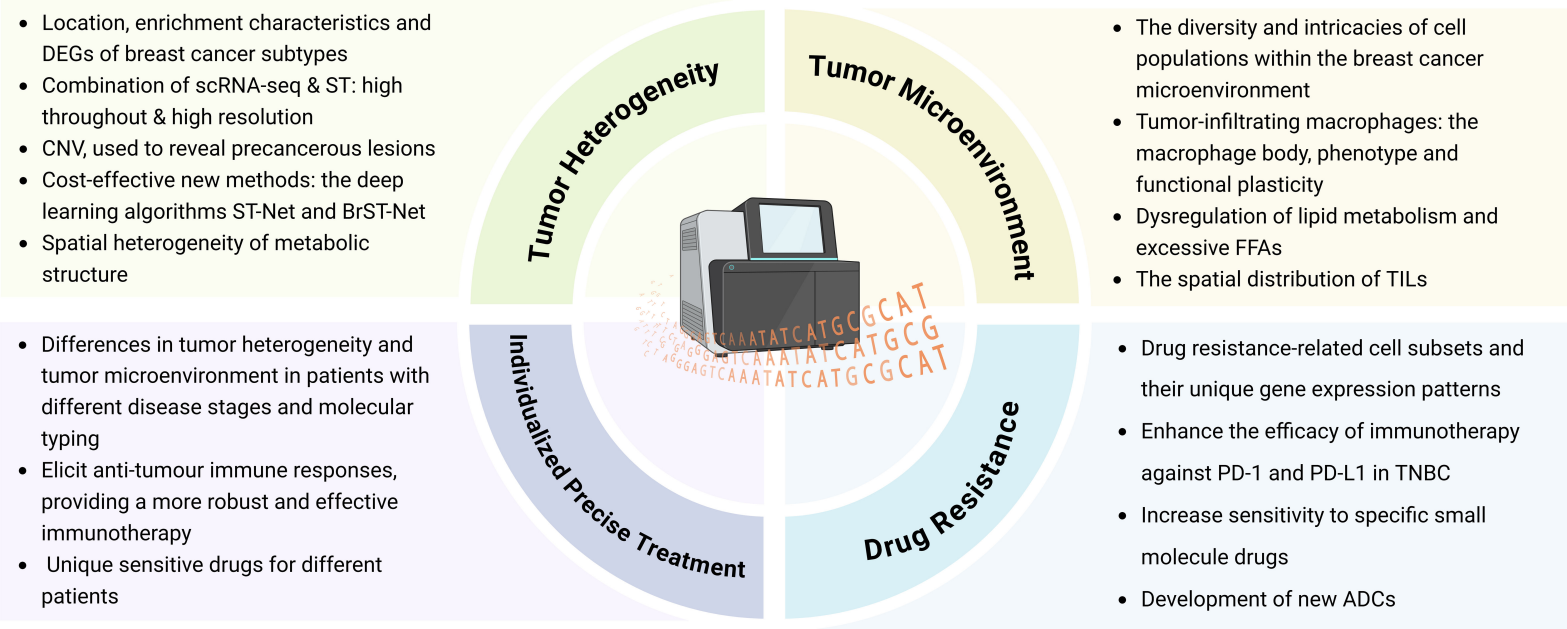
Application of ST in the field of breast cancer research. (Created with BioRender.com).

In conclusion, as shown in **Figure 3**, the integration of ST into basic and translational research can facilitate the development of new drugs by identifying potential therapeutic targets, reveal promising biomarkers to monitor therapeutic effects and guide treatment decisions, and predict unique sensitive drugs for different patients, which is of great significance for promoting individualized precise treatment of breast cancer.

The application of ST in BC treatment provides new perspectives for understanding the TME and individualized treatment, but whether the results are statistically significant or not relies on multifactorial analysis and clinical validation. Currently, ST is still in the exploratory stage, but some preliminary studies have demonstrated its potential in breast cancer and provided statistical significance to some extent. At present, the high cost of ST technology itself and the complexity of data processing also limit its widespread application in clinical settings. Therefore, future studies need to further optimize the technology, expand the sample size and combine it with other histological data (*e.g.* proteomics, metabolomics, *etc.*) for a comprehensive analysis to ensure the statistical significance and usefulness of its clinical application.

## 6 Conclusion and discussion

ST is a pioneering technology used to investigate the heterogeneity of tumors and their microenvironment. It can identify and differentiate the expression of functional genes in specific spatial locations, elucidate the mechanisms, potential drug targets, and novel biomarkers associated with the origin and progression of tumor cell heterogeneity, and investigate the interactions between diverse tumor cell types and immune cells, interstitial components (133–135). ST combines the advantages of traditional transcriptomics and spatial histology to capture the distributional features of genes in tissues without disrupting the tissue structure (136). Key advances in this technology include high-throughput spatially localized RNA sequence analysis, precise mapping of transcript distribution through high-resolution microscopy and imaging, and large-scale spatial transcriptome data acquisition through novel microarray platforms (137).

Initial ST technologies relied on combining tissue slices with microarray platforms by immobilizing RNA on tissue slices and analyzing them using high-throughput sequencing techniques. However, this approach had limited spatial resolution. As the technology has evolved, ST with single-cell resolution has gradually emerged, allowing precise localization of gene expression in individual cells by integrating high-resolution microscopy and genomics approaches (138, 139). In recent years, innovations based on new platforms such as nanopore technology, microfluidic chips and fluorescence imaging have led to more efficient and precise data acquisition of spatial transcriptomes and greatly improved spatial resolution (140, 141). In addition, advances in data processing technologies, such as the application of deep learning and machine learning algorithms, have helped to better analyze complex spatial transcriptome data, revealing cellular interactions and spatial heterogeneity. Nevertheless, despite the rapid advancement of this field, certain limitations persist.

Firstly, the current ST technology is unable to reach the single-cell level in spatial resolution, and the efficiency of gene detection is also low (142). The imaging-based ST technology is subject to limitations, including optical crowding, which restricts the number of genes that can be distinguished. Furthermore, the ST technology of *in situ* capture and sequencing encounters challenges in achieving genuine separation and analysis between single cells, due to factors such as varying capture point resolutions and potential transcript diffusion. The current commercial ST technology has not yet achieved single-cell resolution, and is therefore reliant on bioinformatics algorithms to predict cell types in specific regions (143).

Secondly, the sample data set obtained by ST is not comprehensive enough, which may be due to the differences in sections from different directions and levels. The majority of current ST techniques are based on thin tissue sections, which obtain the expression of genes in two-dimensional space, and still cannot achieve a truly three-dimensional ST. The primary technical challenge lies in developing methods to make the tissue transparent and image the signal within it, or to introduce an imaging probe into the tissue for reaction.

Furthermore, the economic costs and operational complexity also limit the wide application of ST. On the one hand, data processing is a significant obstacle. The complexity, dimensionality, and scale of ST data require the support of powerful computational platforms and algorithms to extract meaningful biological information from large amounts of data. Existing data processing tools and algorithms often struggle to effectively deal with noise, low signal-to-noise ratios, and non-homogeneity of spatial information in spatial data, which limits the effectiveness and feasibility of ST techniques in a wide range of applications (144, 145). On the other hand, ST is costly, especially in the stages of sample preparation, data acquisition and subsequent analysis. High-quality spatial transcriptome experiments usually require expensive equipment (*e.g.* high-resolution microscopes) as well as the consumption of a large number of reagents, which makes the popularization of the technology face financial barriers (146). Finally, the problem of preservation of tissue samples is also a bottleneck limiting the development of ST technology. Most tissue samples need to be processed and analyzed within a short period of time, and prolonged cryopreservation can lead to RNA degradation, which affects the quality of the data (147).

Finally, ST technology is still in the stage of continuous development, and although it has made significant progress in the field of basic research, its maturity is still low. Currently, the application of spatial transcriptome technology is mainly focused on gene expression analysis at the cellular and tissue level, and has not yet been widely used in clinical practice (148). The shortcomings of this technology include the lack of a unified standardized and regulated process, poor data comparability between different platforms, and complex and demanding experimental operations. In particular, no uniform operational specifications have been developed for sample preparation, data collection, and analysis, leading to problems with the reproducibility and accuracy of the technology. In addition, although ST has been significantly improved in terms of resolution and coverage, there is still a need to improve the spatial resolution, especially the precise expression localization at the single-cell level, in order to better reveal cellular heterogeneity and microenvironmental changes. These limitations directly affect the results of ST. In terms of data processing, the complex amount of data and the high-dimensional nature of spatial information may lead to the inability of researchers to accurately capture certain subtle biological features, affecting the interpretation of results (149). Cost constraints, on the other hand, make it difficult for many small and medium-sized laboratories or clinical institutions to afford large-scale ST experiments, further limiting the popularity and scope of application of the technology. Tissue preservation problems may prevent the timely processing of certain samples with valuable biological information, thus affecting the comprehensiveness and accuracy of the study.

Nevertheless, there are multiple innovative techniques that have the potential to overcome these challenges in the future. Firstly, improvements in imaging technologies, especially advances in super-resolution microimaging, can increase the spatial resolution and thus enhance the sensitivity and accuracy of ST techniques (150). Secondly, the application of new computational methods, such as deep learning and artificial intelligence algorithms, is expected to effectively deal with the complexity in ST data, reduce the noise, extract valuable biological information, and help researchers to mine more potential disease biomarkers from the huge amount of data. For the cost issue, the development of automated platforms can improve the efficiency of experiments and reduce the cost of a single experiment, thus promoting the popularity of ST technology. In addition, innovations in molecular preservation technologies may solve the problem of RNA degradation in tissue preservation and improve the reproducibility and stability of spatial transcriptome data (151).

Future research should focus on the following areas: first, improving the efficiency of spatial resolution and data processing, especially in multi-scale ST, how to process cellular to tissue-level data simultaneously; second, developing standardized and automated experimental processes applicable to clinical samples to reduce experimental costs and improve data reproducibility; third, exploring the combination of ST with other high-throughput genomics technologies (*e.g.*, single-cell genomics, metabolomics) to build a more integrated multi-omics platform to comprehensively reveal biological processes (152–154).

The potential applications of ST in many types of cancer are beginning to emerge, particularly in elucidating the TME and cell-cell interactions. In addition to breast cancer, ST has demonstrated great research value in several cancer types, including lung cancer, colorectal cancer, gastric cancer and brain tumors (155–157). In lung cancer, ST has provided important insights into the TME, macrophage reprogramming, immune checkpoints and immunosuppressive changes in brain metastases, providing potential prognostic biomarkers and therapeutic strategies targeting immune and fibrotic pathways (158, 159). In colorectal cancer, on the other hand, ST has helped to investigate genetic and epigenetic co-evolution in colorectal cancer, revealing the influence of chromatin modifications and transcription factors on tumor progression and metastasis (160, 161). The cellular composition and interactions in the TME have been described in detail, identifying key immune cell and fibroblast subpopulations that play important roles in tumor progression, metastasis and prognosis, providing new targets for precision therapy (162). In gastric cancer, ST can help to study the biological properties of gastrointestinal tract tumors at different tissue levels, reveal the gene expression differences between tumors and adjacent normal tissues, and provide important information for early diagnosis and prediction of tumor recurrence (163, 164). And in brain tumors (*e.g.* glioblastoma), ST can help to deeply analyze the cellular heterogeneity in different regions of the tumor, and explore how the tumor cells interact with the surrounding normal neural tissue, so as to discover new therapeutic targets (165).

In addition to cancer, ST has shown immense potential in the study of other diseases (166–168). For example, in neurodegenerative diseases such as Alzheimer’s and Parkinson’s, ST can help study gene expression patterns in different regions of the brain, revealing the spatial heterogeneity of neurodegeneration and key biomarkers (169). In cardiovascular disease research, ST can analyze cell-to-cell interactions in heart tissue, uncovering the spatiotemporal dynamics of cell recovery after myocardial injury, thus providing new therapeutic strategies for heart repair and regeneration (170, 171). Furthermore, in the study of immune-related diseases and metabolic disorders such as diabetes, ST allows for a detailed analysis of cellular composition and gene expression changes in diseased tissues, offering crucial insights into disease mechanisms and precision therapies.

To translate ST technology into clinical practice, the next key step is to achieve its high-throughput and cost-effective standardized application (172). This can be accomplished by developing an ST-based diagnostic platform and integrating it with existing clinical pathology evaluation methods, thereby enhancing the accuracy of early disease diagnosis. Additionally, for research on specific diseases such as cancer and neurodegenerative diseases, greater emphasis should be placed on integrating ST with clinical trials to assess its potential in personalized treatment. Through these practical measures, ST will be able to overcome current limitations and advance the development of precision medicine.

In summary, ST offers the potential to elucidate the spatial information of gene expression with different precision, thereby opening avenues for investigating tumor heterogeneity and TME, and overcoming tumor drug resistance. It is of great importance to identify new biomarkers, study new therapeutic targets and improve the prediction of tumor prognosis. The wide application of ST can improve our comprehension of breast cancer and facilitate the implementation of personalized and precise therapeutic strategies for patients, which will have a profound impact on the field of breast cancer research.
