# Supplementary material for: Spatial transcriptomics in breast cancer: providing insight into tumor heterogeneity and promoting individualized therapy
Source: Front Immunol. 2024 Dec 19;15:1499301. doi: 10.3389/fimmu.2024.1499301 (PMC11693744; doi:10.3389/fimmu.2024.1499301)
Supplement: Supplementary file 1 [file Table1.docx]

**Table S1. Glossary of key terms.**

| **Term** |  | **Definition** |
| --- | --- | --- |
| Convolutional neural network | CNN | A deep learning model automatically learns to extract features from data, particularly images, using convolutional layers to detect patterns and make predictions. |
| Deep learning-based spatio-temporal modeling | DeepST | A deep learning framework for spatiotemporal prediction, specifically designed to predict dynamic spatiotemporal patterns in time series. |
| Digital spatial profiling | DSP | A technique used to measure the abundance of biomarkers (e.g., RNA, proteins) in tissue samples with spatial context, typically employing oligo-tagged antibodies or RNA probes for high-throughput analysis across tissue sections. |
| DNA nanospheres | DNB | A nanostructure made of DNA molecules designed to interact with biological systems, often used for targeting, delivery, or molecular recognition in nanomedicine. |
| Expression-morphology | EMO | Refers to the study of the spatial relationship between gene expression patterns and cellular or tissue morphology, combining transcriptomic data with tissue architecture. |
| Fluorescently-labeled RNA sequences | FISH | RNA molecules that are tagged with fluorescent markers to visualize and track their presence, localization, and expression levels in cells or tissue samples. |
| Geographical position sequencing | GEO‑seq | A spatial transcriptomics technique where RNA sequencing is combined with tissue location data to map gene expression to specific positions in tissue or organs, revealing spatially resolved transcriptomic data. |
| High definition spatial transcriptomics | HDST | A refined version of spatial transcriptomics that offers high-resolution, detailed mapping of RNA expression within tissues, enabling precise localization of gene activity in tissue architecture. |
| High-throughput sequencing, Next-generation sequencing | HTS, NGS | A modern sequencing technique that allows for the rapid sequencing of large amounts of DNA or RNA in a parallel manner, revolutionizing genomics and transcriptomics research. |
| In situ hybridization | ISH | A technique used to detect specific nucleic acid sequences in tissue sections or cell preparations by using complementary labeled probes, often for visualizing gene expression patterns. |
| In situ sequencing | ISS | A method for sequencing RNA or DNA directly within cells or tissue samples, combining the power of sequencing with spatial localization, often used in spatial transcriptomics. |
| Laser capture microdissection | LCM | A technique that uses a laser to isolate specific cells or regions from tissue sections for downstream analysis, allowing for the study of gene expression from carefully selected areas of a tissue sample. |
| Lock-probe in situ sequencing | Lock-seq | A variation of in situ sequencing where the RNA probe is "locked" or stabilized, allowing for more accurate sequence capture and localization within the tissue sample. |
| Model inference and statistical testing for multi-dimensional data | MISTy | A statistical modeling framework is used for inference and hypothesis testing in complex, multi-dimensional datasets, often applied in bioinformatics and other fields requiring high-dimensional analysis. |
| Starfysh | Starfysh | A computational framework designed to analyze brain imaging data and study neural connectivity using advanced neuroimaging techniques. |
| Optimal cutting temperature compound | OCT | A compound used to freeze tissue samples, typically in preparation for cryosectioning, to maintain the tissue's integrity for microscopic analysis and other molecular techniques. |
| RNA tomography | tomo-seq | A computational approach that reconstructs three-dimensional RNA expression patterns within a tissue, often using data from spatial transcriptomics or other imaging-based techniques. |
| Single-cell transcriptomics sequencing | scRNA-seq | A technique for profiling gene expression at the single-cell level, enabling the study of heterogeneity in cell populations by measuring mRNA content in individual cells. |
| Single-molecule RNA fluorescence in situ hybridization | smFISH | A method that detects and quantifies single RNA molecules in cells using fluorescence probes, offering high spatial resolution to track gene expression at the individual molecule level. |
| Spatial transcriptomics | ST | A field of research that combines transcriptomics and spatial biology to map gene expression across different regions of tissue, allowing for an understanding of how gene activity correlates with tissue structure. |
| Standardization in R | StandR | An R package is designed to standardize and harmonize data across multiple sources, facilitating consistent analysis and comparison in statistical modeling and data science. |
| Starfysh | Starfysh | A computational framework designed to analyze brain imaging data and study neural connectivity using advanced neuroimaging techniques. |
| Transcriptome in vivo analysis | TIVA | The study of gene expression within living organisms, typically using high-throughput RNA sequencing techniques to analyze the transcriptomic landscape in various biological contexts. |
